# Surface Appendages of Archaea: Structure, Function, Genetics and Assembly

**DOI:** 10.3390/life3010086

**Published:** 2013-01-24

**Authors:** Ken F. Jarrell, Yan Ding, Divya B. Nair, Sarah Siu

**Affiliations:** Department of Biomedical and Molecular Sciences, Queen's University, Kingston Ontario, K7L 3N6, Canada; E-Mails: yan.ding@queensu.ca (Y.D.); 7ndb@queensu.ca (D.B.N.); 7ss51@queensu.ca (S.S.)

**Keywords:** archaella, archaeal flagella, archaella, type IV pili, hami, cannulae, bindosome, glycosylation

## Abstract

Organisms representing diverse subgroupings of the Domain Archaea are known to possess unusual surface structures. These can include ones unique to Archaea such as cannulae and hami as well as archaella (archaeal flagella) and various types of pili that superficially resemble their namesakes in Bacteria, although with significant differences. Major advances have occurred particularly in the study of archaella and pili using model organisms with recently developed advanced genetic tools. There is common use of a type IV pili-model of assembly for several archaeal surface structures including archaella, certain pili and sugar binding structures termed bindosomes. In addition, there are widespread posttranslational modifications of archaellins and pilins with N-linked glycans, with some containing novel sugars. Archaeal surface structures are involved in such diverse functions as swimming, attachment to surfaces, cell to cell contact resulting in genetic transfer, biofilm formation, and possible intercellular communication. Sometimes functions are co-dependent on other surface structures. These structures and the regulation of their assembly are important features that allow various Archaea, including thermoacidophilic, hyperthermophilic, halophilic, and anaerobic ones, to survive and thrive in the extreme environments that are commonly inhabited by members of this domain.

## 1. Introduction

The study of Archaea has led to great advancements in many fields of biology [1,2], with discoveries that have aided the understanding of processes common to Eucarya and/or Bacteria. In addition, other findings resulted in reports that highlight the novelty of the organisms representing the third Domain of life, such as their unusual and often unique surface appendages [3,4,5,6]. Like their bacterial counterparts, archaeal cells can possess a variety of surface structures that are critical in many aspects of their interactions with the environment. These structures are either (a) entirely unique to the Domain Archaea with no equivalent in either of the other Domains (*i.e.*, hami [7] and cannulae [8]) (b) similar to known bacterial structures but with archaeal-specific twists (pili [9,10,11]) or (c) they are structures which only superficially resemble appendages found in the bacterial domain with fundamental variations (archaella, formerly known as archaeal flagella [12,13,14,15,16,17]). Throughout this review, we will use the term archaellum which has been proposed as a new designation for the structure formerly called the archaeal flagellum. This designation was suggested since the archaeal structure does not resemble the bacterial flagellum in structure or assembly although both function in swimming. Discussion of the merits of the new term within the scientific community continues [18,19].

**Figure 1:**
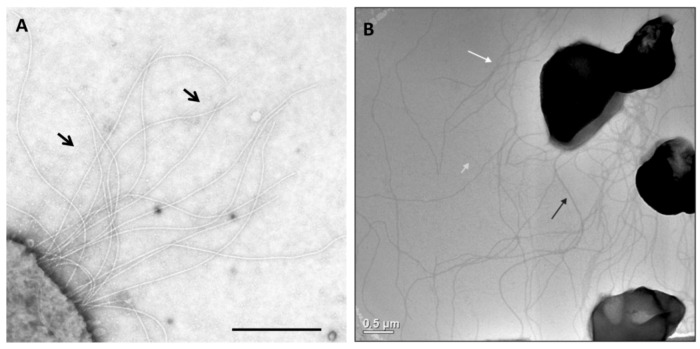
Appendages on the well-studied Archaea *M. maripaludis* and *S. acidocaldarius*. (**A**) Electron micrograph of *M. maripaludis* showing thin pili (arrows) with thicker and more numerous archaella. Bar = 0.5 µm. Courtesy of S.I. Aizawa. Prefectural University of Hiroshima, Japan. (**B**) Electron micrograph of *S. acidocaldarius* showing the presence of three different appendages namely archaella (14nm diameter, black arrow), Aap pili (10–12 nm, white arrow) and threads (5 nm, grey arrow). Bar = 0.5 µm. Courtesy of A.-L. Henche and S.V. Albers, Max Planck Institute for Terrestrial Microbiology, Marburg Germany.

Given the limited number of Archaea for which tractable genetic tools are available [20], it is no surprise that the studies of archaeal surface appendages are limited in most instances to a few model organisms, such as *Halobacterium, Haloferax, Sulfolobus* and *Methanococcus*. Most recently, structural and genetic studies of pili and archaella in Archaea have focused on *Methanococcus maripaludis* and *Sulfolobus* species like *S. acidocaldarius* (Figure 1). The appearance of hami and cannulae is limited thus far to reports in a single genus that lacks genetic systems. Thus data on these unique structures are confined to biochemical and physiological analyses coupled with exquisite electron microscopic studies.

Genetic studies have revealed a preference of Archaea to utilize a bacterial type IV pili model to assemble many of their surface appendages, although the structures formed from these subunits are themselves unique [9,10,11,21]. This assembly mechanism is characterized, among other things, by the presence of class III signal peptides on the major structural proteins, requiring their removal by a dedicated signal peptidase [22,23,24,25,26]. In addition, another very common feature of the major structural subunits of various archaeal surface appendages is attachment of glycan, with N-linked glycan attachment being the best studied [5,8,9,27,28,29,30]. 

Interactions of archaeal cells with their environment through their surface appendages can include such functions as swimming, swarming, attachment to abiotic and biotic surfaces, aggregation, intercellular communication, DNA uptake, virus attachment, nutrient uptake, and biofilm formation. Functional analysis of the various archaeal appendages has confirmed their role in many of these important processes and often multiple functions have been attributed to a single structure or the co-operation of more than one structure is needed in carrying out a particular function [7,9,26,31,32,33,34,35,36,37,38]. The first reports of regulation of surface structure biosynthesis [39] indicate that, in *S. acidocaldarius*, regulation of archaella and pili biosynthesis are linked allowing the cells to adapt to changing environments by simultaneously increasing expression of one structure while repressing synthesis of the other. 

In addition to genetic analysis of several archaeal appendages, several diverse archaeal surface structures (archaella, pili and Iho670 fibres) with the common type IV pili-like assembly model, have now been studied by various electron microscopic and imaging techniques revealing in all cases unusual and unique features [9,10,11,21,40]. 

In this contribution, we have reviewed the available data on surface structures from various Archaea from structural, functional, genetic and assembly aspects. 

## 2. Widespread Use of the Bacterial Type IV Pili System for Archaeal Surface Structures

Archaeal type IV pilus-like structures include archaella [12,17,41,42], type IV-like pili [9,28,34,36], the sugar-binding structure termed the bindosome [43], and the unusual, brittle Iho670 fibers from *Ignicoccus hospitalis* [10] . Bacterial type IV pilins are synthesized as precursor proteins with a class III signal peptide, which is cleaved by a prepilin peptidase (PilD in *Pseudomonas aeruginosa*) [44]. Unlike signal peptidase I or II, whose cleavage site is on the periplasmic side of the cytoplasmic membrane, PilD cleaves the signal peptide from the cytoplasmic side, leaving the hydrophobic N terminal α-helix as part of the mature pilin. Similarly, structural proteins in archaeal type IV pilus-like structures are also synthesized as precursor proteins with usually short signal peptides (often 6–12 amino acids in length; Table 1) and processed by a unique signal peptidase homologous to the bacterial prepilin peptidase [22,23,25,45].

**Table 1:** Seleted archaeal proteins with type IV prepilin-like signal petides.

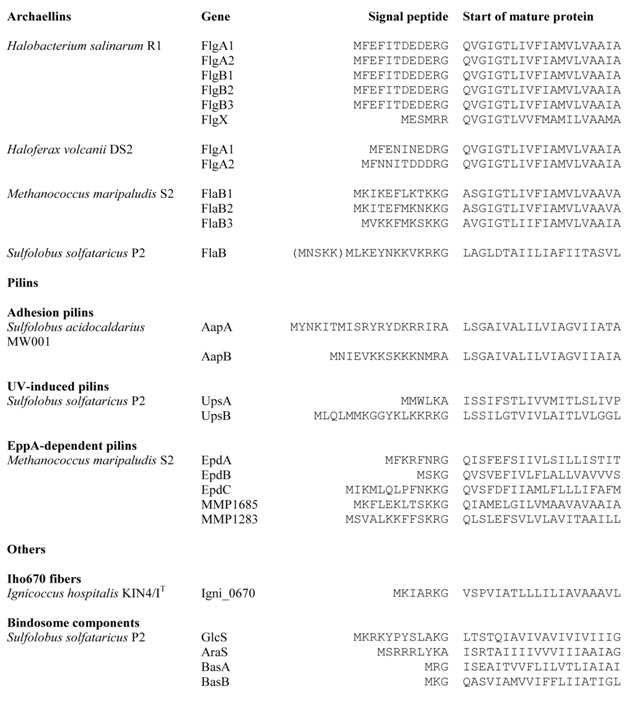

The various archaeal type IV pili-like systems also share homologues of ATPases and a conserved cytoplasmic membrane protein needed for assembly with the bacterial system [46,47]. 

## 3. Archaella

The first appendage in Archaea that was hypothesized to use the type IV pilus-like assembly mechanism was the archaellum [42,48] and it remains the best-studied archaeal type IV pilus-like appendage. Archaella are found commonly on the surfaces of diverse Archaea including methanogens, extreme halophiles, thermoacidophiles and hyperthermophiles [49]. The number and location of archaella varies enormously among different species [49]. Archaella diameters are typically between 10-14 nm [49], although thicker filaments have been reported [50]. Although the major function of this appendage is swimming via filament rotation, like the bacterial flagellum, its structure and assembly are remarkably much more closely related to type IV pili [5,15,16,17,49,51]. 

### 3.1. Fla Operon–Genetic Location of Archaellar Associated Genes

#### 3.1.1. Archaellins

**Figure 2:**
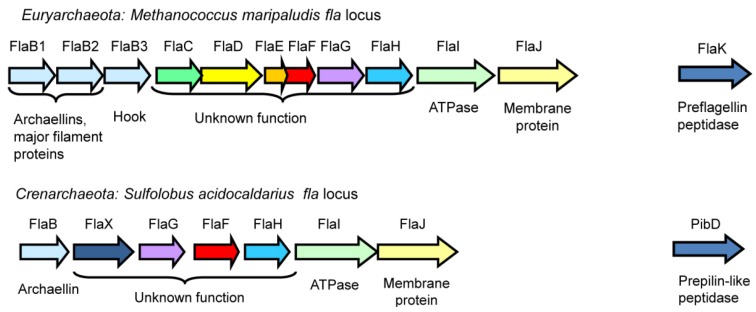
The *fla* operons responsible for archaella formation in a representative euryarchaeote, *M. maripaludis,* and crenarchaeote *S. acidocaldarius*. Homologues in the two systems are shown in identical colours. The prepilin peptidase-like enzymes, typically located outside the operon, are also shown (FlaK and PibD).

In Archaea, most genes involved in archaellation are typically found clustered in the *fla* operon, which usually begins with several archaellin genes (*flaA* and/or *flaB)* encoding the major filament structural proteins, followed by either a complete set of *fla*-associated genes *flaC* to *J* (Euryarchaeaota) or a subset of these genes (Crenarchaeota), as shown in Figure 2 [15]. Genetic studies show that all of the *fla*-associated genes successfully deleted are essential for archaellation [17,52,53]. While some of the *fla* associated genes (*flaI*, *flaJ* and *flaK*) are homologues of genes found in the type IV pili systems of bacteria, others appear to be archaeal specific with no homologues in the bacterial domain [15]. Genes required for posttranslational modification of the archaellin structural proteins (*i.e.* the *agl* genes for N-linked glycosylation, some of which are essential for archaella formation) are located elsewhere in the genome, sometimes scattered in several loci [27,54,55,56].

The first archaellin genes were identified in *Halobacterium salinarum* where five archaellin genes are located in two operons: *flgA1*-*A2* in locus A, and *flgB1-B3* in locus B, while the *fla*-associated genes are transcribed separately [57,58]. All five gene products were identified in archaella samples. Later, complete genome analysis revealed the presence of a predicted sixth archaellin gene, designated *flgX*, located at a distance from the other *flg* genes. The two archaellins in locus A are sufficient to form archaellar filaments [59]. In another halophile, *Haloferax volcanii*, genes encoding the major archaellin FlgA1 and minor archaellin FlgA2 are also transcribed separately from the *fla* associated genes [26]. In *Haloarcula marismortui*, two archaellin genes are found but one is on the chromosome and the second on a plasmid. In different phenotypes of *H. marismortui*, either archaellin can be the major filament protein, with the other found only in very minor amounts [50]. In *Methanococcus voltae*, the major archaellins FlaB1, FlaB2 and the minor archaellin FlaA compose the archaellar filament and the minor archaellin FlaB3 forms the hook region [60,61]. The gene for *flaA* is located immediately upstream of the *fla* operon and transcribed separately [60]. In a related methanogen, *M. maripaludis *S2, the archaellar filament is composed of the major archaellins FlaB1 and FlaB2, while FlaB3 is responsible for the hook region [52]. Deletion of *flaB3* in *M. maripaludis* resulted in functional, but hookless, archaellar filaments [52,61], consistent with the hypothesis that filament assembly occurs before addition of hook subunits at the base (opposite of the bacterial flagella assembly mechanism [62,63]). Interestingly, in the genome sequences of three other *M. maripaludis* strains (C5, C6 and C7), there is a fourth, much longer, archaellin gene in the *fla* operon. The short hook-like filaments in mutant *H. salinarum* cells are also comprised of an archaellin [64]. However, there are many other Archaea in which archaellar hook regions have never been observed, including species that carry a single archaellin gene [12,50]. There are only rare occasions where Archaea with a single archaellin gene have been shown to be archaellated. Studied examples include *Sulfolobus* species [25] and most recently *Halorubrum lacusprofundi* [65]*, *proving that functional helical archaella could be formed from a single archaellin.

#### 3.1.2. FlaCDEFG

In *H. salinarum*, FlaC and E are fused as one protein FlaCE. FlaCE/FlaD proteins were found to have indirect interactions with the chemotaxis proteins via three new identified proteins, indicating that FlaCE/FlaD might be involved in the switch of archaella motor (see below) [66]. In *M. voltae*, FlaC, FlaD and FlaE are all membrane proteins with as yet unidentified functions. Interestingly, both FlaD and a truncated C-terminal version of FlaD have been found in *M. voltae*, *M. maripaludis *and *Methanocaldococcus jannaschii*. The truncated version has high sequence similarity to FlaE [52,67,68]. In *M. maripaludis*, deletions of *flaC* are nonarchaellated; however, deletions of *flaD* or *flaE* could not be created [52]. In light of the halophile evidence, these mutants could be very exciting as they might still assemble archaella but be impaired in rotation switching.

In Crenarchaeota, *flaCDE* are missing, but *flaX, *absent from Euryarchaeota *fla* operons, is present. Switching of rotation of archaella has not been reported in crenarchaeotes, which would be consistent with a role for *flaCDE* in motor switching in euryarchaeotes. Bioinformatics analysis shows that regions in FlaX are homologous with methyl-accepting proteins, implying this protein might be involved in signal transduction [16]. In *S. acidocaldarius*, FlaX is essential for archaella assembly, and FlaH, FlaI and FlaJ are needed to maintain the stability of this protein, suggesting an interaction between these proteins [17]. Recently, the interaction between FlaX and FlaI, the ATPase motor, was confirmed. FlaX forms ring-like oligomers with a diameter of ~30 nm, which is about twice the diameter of the archaellar filament (~14 nm [4]). Both functions are dependent on the presence of the C terminus of FlaX [69].

So far little is known about FlaF and FlaG except the fact that they are essential for archaellation [17,52,68].

#### 3.1.3. FlaHIJ

FlaH, -I and -J are thought to form a secretory complex in archaella assembly [16,68]. *In silico* analysis shows that FlaH is a potential ATPase-like protein containing a typical Walker A motif but an incomplete Walker B motif, which begs the question whether FlaH actually has ATPase function [16]. So far direct biochemical data addressing this issue is lacking [16]. FlaJ is predicted to be an integral membrane protein containing seven to nine transmembrane domains with two highly charged cytoplasmic loops [16,68], and likely forms a crucial component of the central core complex for archaella assembly [16]. It is a homologue of the conserved membrane component of bacterial type IV pili systems (PilC in *P. aeruginosa*) [47] and likely interacts directly with FlaI [16]. 

FlaI belongs to the “secretion superfamily ATPase” or “T2S/T4S ATPase” family involved in bacterial type II secretion, type IV secretion and type IV pili assembly, as well as archaella assembly [47]. Numerous conserved motifs (such as Walker A and B boxes and P-loop motifs involved in nucleotide binding; aspartate box and histidine box) are shared by members of this superfamily. 

Recently, detailed biochemical studies on FlaI from *S. acidocaldarius* were published [70]. FlaI was shown to be a Mn^2+^-dependent ATPase with an optimum pH of 6.5 and temperature of 75 °C. Mutations of key amino acids in the conserved motifs reveal that the Walker A motif is involved in ATP binding, and the Walker B motif is involved in ATP hydrolysis. FlaI ATPase activity is strongly activated by archaeal tetraether lipids but not *E. coli* lipid extracts. FlaI also undergoes an ATP-dependent hexamerization in solution. It is still unclear whether the energy generated by FlaI is used for the archaella assembly by archaellin translocation, or for driving the archaella rotation, or even for both [70].

### 3.2. FlaK-Signal Peptidase for Archaellin Maturation

Archaellins are synthesized as preproteins with a short signal peptide similar to bacterial type IV pilins [22,23,71,72]. Genetic studies show that the removal of the signal peptide is essential for the archaella assembly [23,26]. The most extensively studied archaeal type IV prepilin-like peptidases are FlaK in *M. voltae *and *M. maripaludis* [22,23,73] and PibD in *Sulfolobus solfataricus* [25,72]. Site-directed mutagenesis studies revealed that FlaK/PibD belong to an unusual family of aspartic acid proteases in which two aspartic acid residues, one located within a conserved GxGD motif, are critical for the peptidase activity [23,72,73]. The recently solved *M. maripaludis* FlaK crystal structure confirmed the presence of six transmembrane helices and demonstrated that the enzyme must undergo a conformational change in order to bring the two catalytic aspartic acid residues, located in transmembrane helix 1 and 4, into close proximity [73] . 

The typical length of the signal peptide on archaellins is 6–12 amino acids [15]. Site-directed mutagenesis studies investigated the importance of various amino acid positions in the signal peptide of archaellins. The highly conserved glycine at the -1 position (the cleavage site is +1) was shown to be critical for peptidase cleavage, with the usually basic amino acids at positions -2 and -3 and the conserved +3 glycine also playing important roles [74]. Similar studies conducted on the glucose binding protein precursor, a substrate for PibD in *Sulfolobus* indicated PibD was more flexible in accepting amino acid substitutions around the cleavage site [25]. In *M. maripaludis*, FlaK specifically processes pre-archaellins while the type IV prepilins are processed by another type IV prepilin-like peptidase, EppA (see below) [24]. The *Sulfolobus* PibD, on the other hand, has a more flexible substrate diversity, ranging from archaellins and type IV pilins to the sugar binding proteins that comprise the bindosome [25]. Recent study has indicated that PibD can also process the archaellins of *M. voltae* [75] . In that report, PibD was shown to cleave engineered archaellin signal peptides as short as three and four amino acids whereas FlaK needed a minimal signal peptide length of five amino acids for cleavage. This further supports the more flexible nature of the PibD enzyme. Recently, the prepilin peptidase in *Hfx. volcanii*, also designated PibD, was found to be responsible for the processing of both archaellin FlgA2 and two other type IV pilin-like proteins [26] . The signal peptide cleavage sites of selected archaellins are included in Table 1 [5].

### 3.3. N-Glycosylation Modifications of Archaellin

**Figure 3:**
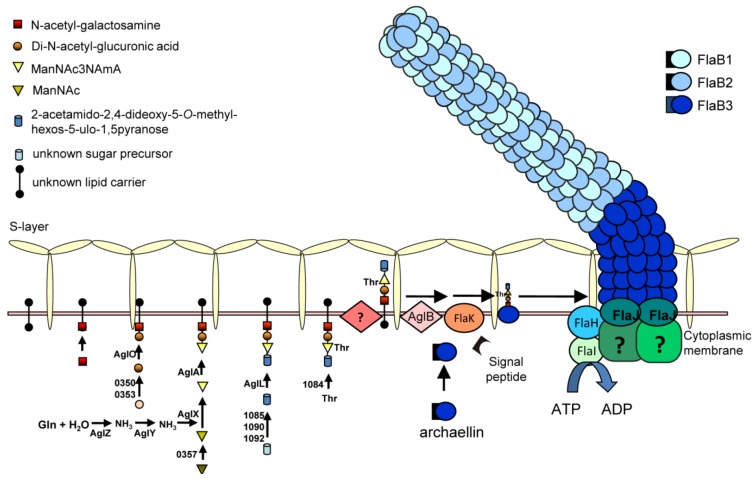
An assembly model for the archaellum of *M. maripaludis*. The N-linked glycan, synthesized by a series of Agl proteins and assembled by Agl glycosyltransferases on an unknown lipid carrier is flipped across the cytoplasmic membrane and attached to the archaellins by the oligosaccharyltransferase AglB. The archaellins are also processed by FlaK which removes the signal peptide and the posttranslationally modified subunits are added to the growing structure by incorporation of new subunits at the base through the activities of the ATPase FlaI in conjunction with the conserved membrane protein FlaJ.

N-glycosylation is a significant and likely widespread post-transcriptional modification for archaellins. All the archaellins in *M. voltae*, *M. maripaludis*, *H. salinarum *and *Hfx. volcanii* are modified with N-linked glycans [29,30,76,77]. Analysis of available archaellin sequences only rarely reveals ones that lack potential N-linked glycosylation sites (such as both archaellins of *H. marismortui* [65]) while some archaellins show a remarkable number of such sites: *Methanothermococcus thermolithotrophicus* FlaB2 has the most with 16 predicted N-glycosylation sites. For most organisms, further work is needed to confirm whether these sequons are actually occupied by N-glycan [15]. Using archaellin as the reporter protein, an N-glycosylation model has been established in *Methanococcus* spp, and its role in the archaellum assembly model in *M. maripaludis* is depicted in Figure 3. In the cytoplasm, the N-glycan precursor is assembled on an unknown lipid carrier by sequential addition of sugar monomers by the corresponding glycosyltransferases, followed by flipping of the glycan across the plasma membrane by an as yet unidentified enzyme. Finally, the N-glycan is transferred *en bloc* to the N-X-S/T (X=/P) motifs in the archaellins by the oligosaccharyltransferase AglB [54,55]. In *M. voltae*, both the archaellins and S-layer protein are N-glycosylated with a trisaccharide β-ManNAcA6Thr-(1–4)-β-GlcNAc3NAcA-(1–3)-β-GlcNAc [54]. AglH and AglA are glycosyltransferases responsible for the 1^st^ and the 3^rd^ sugar, respectively, and AglC and AglK are either the glycosyltransferases or involved in the biosynthesis of the 2nd sugar [54,78,79]. In *M. maripaludis*, the archaellin N-glycan is a tetrasaccharide Sug-4-β-ManNAc3NAmA6Thr-4-β-GlcNAc3NAcA-3-β-GalNAc, where Sug is a diglycoside of an aldulose exclusively found in this species. Glycosyltransferases responsible for the 2nd, 3rd and 4th sugars have been identified as AglO, AglA and AglL, respectively [55]. A number of genes encoding enzymes involved in the biosynthesis of the individual sugar components have also been identified [80,81,82] (Y. Ding, S. Siu and K. Jarrell, unpublished data).

Study of various mutants carrying deletions in *agl* genes that result in truncated glycan have shown that a minimum length of the N-glycan is essential for archaella assembly. In both *M. voltae* and *M. maripaludis*, no archaella were observed on the cell surface when the archaellins were nonglycosylated or carried a glycan that consisted of only a single sugar [54,55], indicating a minimum 2-sugar glycan was necessary for archaellins to assembly into an archaellar filament. When archaellins were modified with a 2- or 3-sugar glycan, the cells assembled archaella but these cells were less motile in swarm plate assays than the wildtype cells that carry the entire 4-sugar glycan [55]. 

In *Hfx. volcanii*, archaellins were recently found to be modified with the same N-glycan originally found decorating the S layer protein [30,83,84,85]. The pentasaccharide is composed of a mannose, a methyl ester of hexuronic acid, two hexuronic acids and a hexose [84,86]. As found in *Methanococcus* species, deletion of the oligosaccharyltransferase gene, *aglB,* in *Hfx. volcanii*, led to non-archaellated cells, while swarm plate assays indicated that attachment of a minimum of a 3-sugar glycan to archaellins is necessary for archaella formation [30]. 

While N-linked glycosylation was shown to be necessary for archaealla formation in *Methanococcus* and *Haloferax*, the archaellins of each species carry multiple N-glycosylation sites. In the case of *Hfx. volcanii* FlgA1 (the major archaellin) all three sites seems to be important in archaella assembly or function since site directed mutagenesis to individually eliminate each site led to non-motility on semi-solid swarm plates [30]. Interestingly, this does not appear to be the case for *M. maripaludis*. In this organism, a spontaneous mutant carrying a FlaB2 version with an asparagine (N) to aspartic acid (D) mutation in the N-X-S/T motif which eliminates the 2^nd^ N-glycosylation site was discovered. This strain however produces functional archaella (Y. Ding and K. Jarrell, in preparation).

### 3.4. Archaella Regulation

Archaella synthesis is known to be regulated in both *M. jannaschii* and *M. maripaludis*, depending on the availability of H_2_, with archaella synthesis induced under H_2_ limitation conditions [67,87]. Further study using quantitative proteomics of nutrient-limited *M. maripaludis* indicated that the expression of archaellins was affected by multiple nutritional factors: decreased under nitrogen limitation but increased under phosphate limitation [88]. It was suggested that *M. maripaludis* may respond to nitrogen limitation conditions by shutting down other energy intensive processes like motility when forced to switch to the energy-consuming nitrogen fixation pathway. In *S. solfataricus*, transcription of the archaellin gene is highly increased in the stationary phase and when the cells encounter nitrogen starvation growth conditions [17].

In the *fla* operon of *S. acidocaldarius*, two differentially regulated promoters have been identified [17,39], one lying upstream of the gene encoding the major structural protein FlaB and a second promoter upstream of *flaX* that regulates transcription of the downstream genes *flaX-J*. Transcriptional readthrough also occurs for the *flaB* gene likely due to a weak termination signal. Under tryptone limiting conditions, the expression of both *flaB* and *flaX* was shown by qRT-PCR to dramatically increase showing that the regulation of archaella synthesis can be observed at the transcriptional level. Promoter studies further revealed that in an Aap pilus minus background, the *flaB* promoter activity was dramatically increased under starvation stress, while no difference was observed with *flaX *promoter. To date, no data has been presented on mRNA stability or protein half-lives but nonetheless the results presented by the Albers group so far suggest that the expression of the archaellar accessory and core components is under the constitutive *flaX *promoter to preserve the core complex for quick archaella assembly, which depends then only on the availability of the major structural protein archaellin. Meanwhile, expression of the energy-consuming archaellin subunits depends on the inducible *flaB *promoter in response to environmental cues, such as starvation [17].

In the methanogens where regulation of archaella has been observed, no transcriptional regulators have been reported. In *S. acidocaldarius*, no activators have been found responsible for the induction of *flaB*, but two repressors of archaellation, ArnA and ArnB, have been identified [39]. ArnA is a forkhead-associated (FHA) domain-containing protein. Typically, FHA-domain containing proteins have phosphopeptide-binding activities. ArnB contains a von Willebrand (vWA) domain and proteins carrying such a domain typically form multi-protein complexes. Indeed, both homologously and heterologously expressed ArnB can be co-purified with a His-tagged version of ArnA and *vice versa*, indicating a strong *in vivo* interaction between these two proteins. Both ArnA and ArnB can be phosphorylated by specific eukaryotic-like protein kinases and dephosphorylated by Ser/Thr phosphatase PPP from the same species *in vitro*. Under tryptone starvation conditions, cells of *ΔarnA* and *ΔarnB* mutants are hypermotile via hyper-archaellation, implying that ArnA and ArnB are repressors for the *fla* operon [39]. While it was expected that ArnA would bind directly to the *flaX* promoter as observed in *S. tokodaii* [89], further studies showed that in *ΔarnA* and *ΔarnB* mutants, the activity of both the *flaB* promoter and *flaX* promoter was not as dramatically upregulated as expected, suggesting that ArnA and ArnB are not acting primarily at the transcriptional level in *S. acidocaldarius* but rather on a protein-protein interaction level. Since Arn homologues are not found in euryarchaeotes, regulation of archaellation in this archaeal group must occur by a different mechanism. Further layers of archaella control are suspected in *S. acidocaldarius*, including a likely positive regulator for the system [39] as well as a role for anti-sense RNAs [90]. 

In *S. acidocaldarius*, two observations point to a regulatory interplay between the archaella and Aap pili systems that might coordinate the expression of different surface appendages depending on different environmental signals. Firstly, expression of FlaB was dramatically induced in the Aap pilus minus mutant [17]. Secondly, overexpression of the archaella repressor ArnA led to hyper-piliation [39]. 

Gene deletions that lead to nonarchaellation of *M. maripaludis* have been reported, including ones in the *fla* operon but also ones affecting glycosylation of the archaellins (*agl* genes) [55]. In such mutants, archaellin structural proteins are not detected after subsequent transfers suggesting that secondary mutations have occurred that have resulted in cessation of transcription of the *fla* operon, presumably resulting in energetic savings under conditions where archaella cannot be assembled. These mutations are not located in the promoter region and may be in genes encoding activators for the *fla* operon (G. Jones and K. Jarrell, unpublished observation).

### 3.5. Archaella Structure

The archaellum has been described as “a bacterial propeller with a pilus-like structure” [40]. Knowledge about archaella structure is very limited, mainly from *H. salinarum* and *Sulfolobus shibatae, *a euryarchaeote and a crenarcheote living in very different extreme environments [21,91,92]. Despite the phylogenetic distance between the two organisms, three dimensional reconstructions of the archaella from the two species are similar in structure and provide a basic symmetry for archaellar filaments that is distinct from bacterial flagella filaments. The outer domain forms a 3-start helix wound around a solid inner core domain, which lacks an internal channel. The inner core domain is conserved both in size and shape, with a diameter of 5 nm in both archaea, and thought to be constructed as alpha-helices by the hydrophobic N-terminal segment of archaellins. The N-terminal sequences of archaellins are highly conserved and homologous to those of type IV pilins [41], where they are known to be involved in subunit-subunit interactions in that bacterial appendage [21]. Considering that mature archaellins have a highly conserved and hydrophobic 30-40 amino acids at the N-terminus [45,71,93], this structure might apply to the whole archaeal domain. Compared with the conserved inner core, the size of the outer domain varies, and is responsible for the differences in the diameter of archaellar filaments in *S. shibatae* (14 nm) and in *H. salinarum *(10 nm). These variable regions might reflect adaptations of the different archaella to optimize their performance to specific harsh environments inhabited by a variety of archaea [21,91]. 

Scanning 10 amino acid deletion analysis of the archaellin FlaB2 in *M. maripaludis *was recently conducted (Y. Ding and K. Jarrell, in preparation). Complementation of a *flaB2* deletion strain with any of the 10 amino acid deletion versions of *flaB2*, including deletions in the variable region of the protein, did not restore archaellation. Complementation with a *flaB2* variant carrying only a three amino acid deletion in the variable region led to only poor restoration of archaellation, suggesting perhaps that absolute length of the archaellin is important for proper archaella assembly.

### 3.6. Archaella Function

The archaellum is a motility apparatus widespread throughout the domain Archaea, and helps archaeal cells swim in liquid medium or swarm through semi-solid medium [17,52,53,94]. The archaellum rotation, like that of bacterial flagella, can be clockwise or counterclockwise, at least in *Halobacterium*, indicating the presence of a switch [95]. In bacteria, the rotation of flagella is under the control of the chemotaxis system and binding of phospho-CheY to the switch protein FliM has been documented [96]. In Archaea, a bacterial-like chemotaxis system including *cheY* homologues has been identified in euryarchaeota [97,98] but not in crenarchaeota [99]. Nevertheless, homologues to FliM have not been reported and where the interaction between the archaella and the chemotaxis system occurs has not been identified [100]. As mentioned, three newly identified proteins encoded by genes close to *fla* operon were found to be the mediator between the chemotaxis proteins CheY, CheD and CheC2 and FlaCE/D in *H. salinarum* [66]. 

Until recently, information on swimming speeds of different Archaea was extremely limited. Cells of *H. salinarum* were reported to swim at speeds of 2-3 μm per second [95] and it was unknown whether such slow speeds were typical of Archaea in general. Recently, using a “thermo-microscope”, the swimming speed of selected species of hyperthermophilic Archaea was measured [101]. If speed is measured in bodies per second (bps), the two hyperthermophilic methanogens *M. jannaschii *and *Methanocaldococcus villosus* are the fastest swimmers so far reported, with speeds of close to 400 and 500 bps (absolute velocity of 468 and 589 µm/s, respectively; compare to *E. coli* speed of 20 bps). Swimming speeds were also measured for numerous other Archaea, including ones where genetic studies on archaellation have been done. These include *M. voltae* (128 µm/s, 64 bps), the weakly motile *M. maripaludis* (45 µm/s, 30 bps) and the thermoacidophile, *S. acidocaldarius* (60 μm/s, 40 bps).

**Figure 4:**
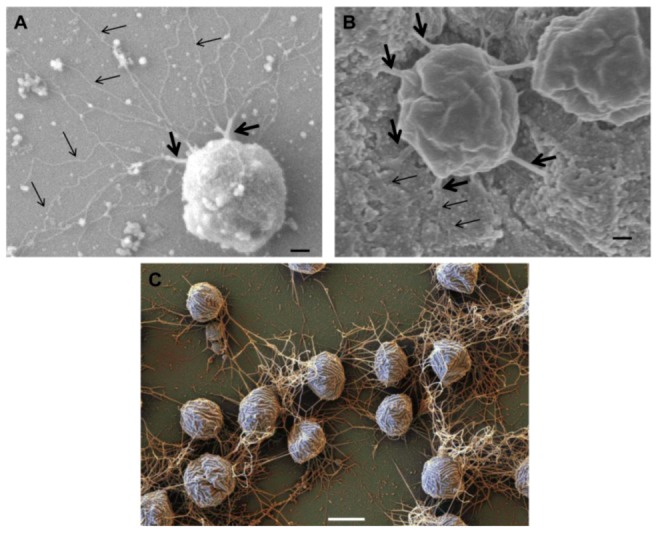
Role of archaella in attachment of Archaea to surfaces and other cells. Scanning electron micrograph of *M. maripaludis* attached to silicon wafer via thick cables of archaella (thick arrows) which can unwind to individual archaellar filaments (thin arrows). Bar = 100 nm (**B**). Connection of *M. maripaludis* cells to each other and underlying nickel EM grid via archaellar bundles. (**A**) and (**B**) reprinted from [31]. Bar = 100 nm (**C**). Scanning electron micrograph showing attachment of *Mcc. villosus *cells to a surface and to other cells via bundles of archaella. Bar = 1 µm. Courtesy of Gerhard Wanner, University of Munich, Germany.

Besides swimming, the archaellum plays critical roles in other functions for Archaea such as surface adhesion and cell-cell contact and, perhaps, even intercellular communication. In *Pyrococcus furiosus*, where the archaellum is the only surface appendage, it is responsible for cellular adhesion to different kinds of abiotic surfaces. Bundles of archaella also form cable-like structures mediating cell-cell contact [102]. Interestingly, P*. furiosus* uses archaella to adhere onto cells of another archaeon, *Methanopyrus kandleri,* to form a bi-species biofilm [37]. Cable-like structures composed of archaella were also shown to mediate cell-cell contact and abiotic surface adhesion in *M. villosus* [103] and *M. maripaludis* [31] (Figure 4).

When *P. furiosus* and *M. villosus* were cocultured, the growth of both archaea increased, and cells of these two species were connected via archaella and formed “flocks” in liquid medium, even without the presence of a solid surface. Although the function played by archaella under this situation is not clear, it was speculated that archaella may mediate signalling and interaction between both partners [38]. Such a precedent was reported in the syntrophic relationship between the methanogen *Methanothermobacter thermoautotrophicus* and the bacterial syntroph *Pelotomaculum thermopropionicum*. Here, the archaeon perceives the flagellar cap protein FliD of the bacterium and up-regulates a number of genes involved in methanogenesis, ATP synthesis and hydrogen utilization, thus preparing itself for the onset of the syntrophic interaction and indicating that cell surface appendages can have critical roles in intercellular communication [104].

In some Archaea, the function of the archaella in adhesion and biofilm formation may be intertwined with other surface structures such as pili. In *M. maripaludis* and *S. solfataricus*, both archaella and pili are necessary for attachment to abiotic surfaces [31,105]. In *M. maripaludis*, bundles of archaella are clearly observed in the persistence stage of adherence and seem to be strong enough to maintain the attachment, which lead to the speculation that pili may play an important role in the initial stages of attachment [31]. However, in *S. acidocaldarius*, although archaella, the UV-induced pili (Ups pili) and the adhesive pili (Aap pili) all play roles in surface adhesion and biofilm formation, archaella appear to have only minor effects compared with the other two appendages, but they are speculated to play a role in cell release from the biofilm [32]. In *S. solfataricus*, the expression of archaellin FlaB was significantly reduced in adherent cells, implying that archaella might play important roles in the initial attachment but not the persistence [105]. In Archaea in which archaella play an important role in persistence of adhesion to surfaces (*P. furiosus, M. villosus* and *M. maripaludis)*, bundles of archaella are commonly observed. In contrast, in *S. acidocaldarius*, archaellar bundles have not been reported, suggesting that archaella in this species are used for motility and attachment initiation, but not for the persistence of attachment [32]. 

## 4. Pili

Structures believed to be pili were observed in electron microscopic studies of various Archaea decades ago [106,107] but it is only recently that studies specifically focused on the structure, assembly, genetics and function of these appendages have been reported. Most of the recent pili studies have focused on the genetically tractable species within the genera *Sulfolobus* and *Methanococcus. *Bacterial pili are involved in many different functions, such as adhesion, twitching motility, DNA uptake, and biofilm formation [108,109]. In Archaea, various pili functions have also been reported including adhesion, cell aggregation, biofilm formation, and DNA exchange [34,36].

**Figure 5:**
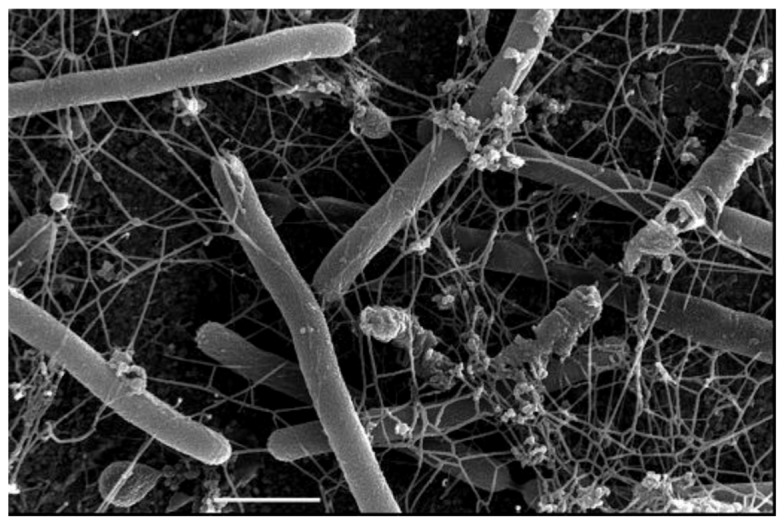
Scanning electron micrograph of *M. thermautotrophicus* grown on gold EM grids and expressing many Mth60 fimbriae. Bar = 1µm. Courtesy of Gerhard Wanner, University of Munich, Germany.

All archaeal pili studied thus far are type IV pili–like with one exception, *i.e.*, the Mth60 fimbriae of *M. thermoautotrophicus* [110]. The Mth60 pili, 5nm in diameter, are composed of a 16kDa glycoprotein with a predicted length of the mature processed pilin of 143 amino acids. While the nature of the glycan attached has not been reported, there are potential N-glycosylation sites present in the protein. There is an interesting regulation in the biosynthesis of the pili since they are barely observed on the surfaces of planktonic cells but found in high numbers when cells were grown on surfaces (Figure 5). The Mth60 pili were the first archaeal pili shown to play a role as adhesins both to abiotic surfaces and to other cells. Interestingly, these pili are a rare case of an archaeal surface structure that can be stained effectively with succinimidyl esters of fluorescent dyes (Alexa dyes) [111]. Recently, another *Methanothermobacter* isolate, *M. tenebrarum*, was reported to possess bundles of polar pili [112]. 

### 4.1. Type IV Pili-Like Loci in Archaea

**Figure 6:**
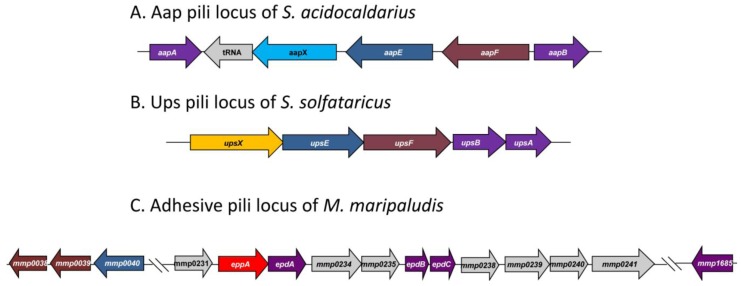
Type IV pili-like loci found in three different Archaea. Functionally similar genes are given identical colors. (**A**) The adhesive pili locus of *S. acidocaldarius *showing the pilin-like genes *aapA/B*, the iron sulfur oxidoreductase (*aapX*), the ATPase (*aapE*) and the inner membrane protein (*aapF*). (**B**) The UV-inducible pili locus of *S. solfataricus *contains genes for two pilin (*upsA* and *upsB*), an ATPase (*upsE*) and a conserved membrane protein (*upsF*) as well as a protein of unknown function* (upsX) *and (**C**) The adhesive pili locus found in *M. maripaludis *contains genes for three minor pilins (*epdA,B,C*), a prepilin peptidase (*eppA*) and a number of proteins of unknown function. Located outside this locus are genes for the ATPase (MMP0040), inner membrane proteins (MMP0038 and MMP0039) and the major pilin gene (MMP1685).

Genetic and structural work on type IV-like pili has been reported in both the crenarchaeota (*Sulfolobus *species) and euryarchaeota (*M. maripaludis*). All *Sulfolobus* species studied (*S. acidocaldarius, S. solfataricus* and *S. tokodaii*) possess UV-inducible pili encoded by the *ups* operon while only *S. acidocaldarius* shows, in addition, the presence of a second type IV pili system termed adhesive (Aap) pili. A comparison of the genetic loci encoding these appendages in different Archaea is presented in Figure 6.

#### 4.1.1. Adhesive (Aap) Pili of *S. acidocaldarius*

The Aap pili locus of *S. acidocaldarius *consists of five genes [9] (Figure 6). A *pilB* ATPase homologue (*aapE*) and a *pilC* integral membrane protein homologue (*aapF*) have been identified in the locus together with *aapX*, a gene encoding an iron-sulfur oxidoreductase. Flanking these genes, but transcribed in the opposite direction are two pilin genes (*aapA* and *aapB*). Mutants containing deletions in genes that prevented both archaellum and Ups pili formation still had pili on their surface. Mass spectrometry of pili, purified from such mutant cells, revealed the major pilin to be encoded by *aapB* [9]. These pilins subunits, like all other preproteins with class III signal peptides in *S. acidocaldarius*, were processed by PibD. Deletion mutants were created in each of the *aap* genes and analyzed for the presence of pili [9]; all five genes were essential for pili biosynthesis. 

#### 4.1.2. UV-Inducible Pili of *Sulfolobus* Species

UV-inducible pili were first reported in *S. solfataricus* [36] where an operon of five genes with a similarity to type IV pili genes was found (Figure 6). The UpsA and UpsB proteins had a class III signal sequence similar to that seen in type IV pilins, and UpsB was shown to be processed by PibD. The signal peptide present on UpsA is too small (see Table 1) to allow separation of the processed from the nonprocessed form in SDS-PAGE in the standard processing assay, although it is likely that UpsA is also a substrate for PibD, since PibD is the only prepilin-like peptidase in this organism. An ATPase (UpsE) and an integral membrane protein (UpsF) which showed similarities to the corresponding proteins, TadA and TadC respectively, in the type IV pili system (Tad pili) of *Aggregatibacter* [36,113] were also identified. The final protein encoded in this locus was a predicted highly hydrophilic protein UpsX that was unique to *Sulfolobales* and its role in Ups pili formation is currently unknown [36]. Deletion analysis demonstrated that the ATPase of the operon was necessary for formation of the pili after UV treatment, linking this operon with the Ups pili structures. Whether UpsA or UpsB is the major structural protein of the pili is unknown. 

The UV-induced pili operon is well conserved in other species of *Sulfolobus*, such as *S. acidocaldarius* and *S. tokodaii* [24]*.*

#### 4.1.3. Pili Locus in *M. maripaludis*

A type IV pili locus, containing 11 potential genes (*mmp0231-mmp0241*) was predicted using bioinformatics in the genome of *M. maripaludis* [24] (Figure 6). This locus is unlike the type IV pili loci in the order *Sulfolobales*, where there are only five genes. The 11 genes form a single transcriptional unit that consists of three pilin-like genes (*epdA, epdB* and *epdC*), a prepilin peptidase (*eppA*) and a number of other genes which have no homologues in either bacterial type IV pili systems or the Aap or Ups pili of *Sulfolobus* species. Some are even restricted to *Methanococcus* species (D. Nair and K. Jarrell, unpublished data). Unlike the *Sulfolobus* pili systems, the genes encoding the major pilin (MMP1685), as well as the ATPase and the conserved pilus membrane protein were not found in the locus. The gene encoding the ATPase necessary for assembly of the *M. maripaludis* pili (*mmp0040*) was identified in a small gene cluster, that also included two genes (*mmp0038* and *mmp0039*) with homologies to the conserved membrane proteins of type IV pili systems (D. Nair and K. Jarrell, in preparation). Also unlike in *Sulfolobus* where all substrates with class III signal peptides are processed by PibD, in *M. maripaludis*, there is a devoted prepilin peptidase, EppA, which only cleaves the prepilins while FlaK processes archaellins [22,23]. EppA is a larger protein with additional four transmembrane segments compared to FlaK [24]. The substrates of EppA have a negatively charged amino acid at the +5 position, similar to most of the bacterial type IV pili and missing in the non-EppA substrates (*i.e.*, archaellins) [5]. By creating a genetic hybrid, where the four amino acids (KGAS) around the cleavage site of FlaB2 (archaellin) were substituted for the cleavage site (RGQI) of EpdA (pilin), the importance of a +1 glutamine for cleavage was shown [24]. 

Deletion analysis of all 11 genes in the original pili locus as well as other type IV pilus gene homologues found outside the locus was conducted. Deletion of most of the genes resulted in a phenotype with no piliation. The three pilin-like genes (*epdA*, *epdB* and *epdC*) [28], the prepilin peptidase (*eppA*), the major pilin protein (*mmp1685*), the ATPase (*mmp0040*), and both conserved membrane proteins (*mmp0038* and *mmp0039*) were all found to be essential for normal piliation (D. Nair and K. Jarrell, in preparation). In addition, some of the other genes in the locus, annotated with unknown function, such as *mmp0234*, *mmp0239*, *mmp0240* and *mmp0241* were also found to be essential for piliation. Repeated efforts to delete *mmp0231* were unsuccessful. The two genes in the cluster that were found not to be essential for pili formation were *mmp0235* and *mmp0238*. *M. maripaludis* pili are known to have a role in attachment on solid surfaces, although only in the presence of archaella [31]. Whether the pili assembled in the absence of MMP0235 or MMP0238 can still function in attachment is currently under investigation. Interestingly, both of these proteins have predicted signal peptides and it is possible that one of them may function as a tip adhesion. 

While studies directed at the biogenesis of archaeal type IV-like pili have not been presented, it seems likely to generally follow the process of bacterial type IV pili. However, the presence of essential genes in both *Sulfolobus* and *Methanococcus, *that have no homologues in bacterial systems, suggests that there will be aspects unique to the archaeal domain. 

### 4.2. Pili Regulation

The regulation of archaeal surface structures is in its infancy and information on the regulation of pili is limited to several recent observations in *Sulfolobus* species. Ups pili were first identified when a putative pilus gene locus was strongly upregulated after cells were UV irradiated. This upregulation was apparently dependent upon DNA double strand breaks as other DNA damaging agents, like bleomycin, had similar effects [36]. Understanding of the molecular mechanism behind the induction is currently not known but very recently it was shown to involve the transcriptional regulator Sa-Lrp in *S. acidocaldarius* [114]. Examination of a strain deleted for Sa-lrp demonstrated that it was defective in the UV-induced aggregation mediated by Ups pili and qRT-PCR confirmed that, in the mutant, *upsA* transcript levels were eight-fold lower than that of wildtype cells following UV induction. It was suggested that Sa-Lrp likely acts in conjunction with other regulators at the transcriptional level [114]. 

Expression of Aap pili in *S. acidocaldarius* is known to be growth phase dependent. Lower numbers of Aap pili are found on cells in stationary phase and qRT-PCR showed reduced levels of transcript for *aap* genes in the stationary phase compared to the exponential phase [9]. Several observations point to an interplay in the regulation of Aap pili and archaella (see Section 3.4.). Archaella are up-regulated in the stationary phase when Aap numbers are reduced. In addition, archaella expression was increased in all *aap* gene deletion strains, especially in the *aapF *deletion strain*,* which was hyper-archaellated [9,17] and finally overexpression of ArnA, a repressor of archaellation leads to increased Aap pili production under tryptone starvation conditions [39]. Additional layers of regulation are likely as numerous antisense RNAs are predicted to lie with *aapF* [90]. However, their actual effect on Aap pili formation has not yet been reported. 

### 4.3. Pili Structure

The first structure of an archaeal pilus was that from *M. maripaludis* [11]. The 6nm diameter pili in this organism are found in small numbers (5–10 per cell) and located peritrichously. Despite the similarities of the *M. maripaludis* pilins to bacterial type IV pilins, cryo-electron microscopy of *M. maripaludis* pili showed a structure that was different from that of any bacterial pili (type IV or otherwise) or archaella. This study showed that two different helical symmetries existed and that they coexisted within the same archaeal pilus [11]. A hollow lumen with a diameter of 20Å was observed, a feature missing in archaella structures [21]. 

Mass spectrometry of purified *M. maripaludis* pili samples identified the major structural protein to be MMP1685, a small glycoprotein of only 74 amino acids including a 12 amino acid class III signal peptide [28]. Interestingly, the glycan N-linked to this pilin was not identical to that previously characterized on archaellins but instead had an additional hexose attached to the linking sugar, GalNAc. The purpose of the added sugar and the identity of the glycosyltransferase responsible for the hexose addition are not known. As found in Aap pili of *S. acidocaldarius,* as well as type IV pili systems of bacteria in general, the structure in *M. maripaludis* is composed of a single major pilin although there is genetic evidence for the role of at least three other proteins as minor pilins [28]. 

Ups pili, with a diameter of 10 nm but of variable lengths, are peritrichously located on the surface of different *Sulfolobus* species [34,36]. The structure of *S. solfataricus* Ups pili showed straight fibers consisting of three evenly spaced helices with a pitch of 15.5 nm. 

Very recently, the structure of another archaeal pilus, the extremely stable *S. acidocaldarius* Aap pili, with a diameter of 11 nm, was published [9]. Remarkably, the Aap pilus structure was also different from known bacterial pili or the *M. maripaludis* pili or indeed any other archaeal type IV pili-like structure including archaella and Iho670 fibers. The Aap pilus displayed a rotation per subunit of 138° and a rise per subunit of 5.7 °, very different from those of studied bacterial type IV pili. These studies indicate that although the building blocks of the many archaeal appendages are similar, the small sequence changes can lead to very different quaternary structures [9,11]. 

### 4.4. Pili Function

Study of archaeal pili has revealed a variety of functions, mainly related to attachment and biofilm formation, sometimes in conjunction with archaella.

Ups pili appearance is correlated with cellular aggregation which enhances the exchange of chromosomal DNA, likely aiding in the population overcoming the DNA damage which leads to Ups pili biosynthesis [36,115]. Support for this comes from studies on mutants unable to make Ups pili. These strains were unable to exchange DNA when UV-induced and this resulted in decreased survival of the cells. Formation of Ups pili by at least one partner is essential for the exchange of DNA. Even though *S. acidocaldarius, S. solfataricus* and *S. tokodaii* are all capable of synthesising UV inducible pili that lead to cell aggregation, the aggregation only occurs between cells of the same species [34] suggesting that there is a specific recognition of the cell surface by the pili. Perhaps different N-linked glycosylation structures on the pilin subunits are involved in this species specific recognition. In addition to their role in cell aggregation and DNA exchange, Ups pili can also play a role in attachment to surfaces but the importance of Ups pili in surface attachment varies among different *Sulfolobus* species. In *S. acidocaldarius*, where both Aap and Ups pili are made, the Ups pili have little effect on surface attachment [32]. However in *S. solfataricus*, Ups pili, in collaboration with archaella, are essential for adherence, since mutants lacking the ability to form either one of the surface structures were unable to attach to a variety of tested surfaces [105]. Analysis of adherent cells by qRT-PCR showed an upregulation of the Ups pilin genes (*upsA* and *upsB*) with a significant decrease in the transcription of the archaellin gene *flaB*, suggesting the role of archaella may be in the initial attachment but not in persistence after attachment. Ups pili in *S. solfataricus* may have a role in biofilm maturation [33,105] while in *S. acidocaldarius,* mutants defective in Ups pili formation have changes in the structure and development of biofilms [32].

The major function of Aap pili is attachment of *S. acidocaldarius* cells to surfaces. Their exceptional stability reflects the need for these structures to function in the extreme thermoacidophilic environments inhabited by *S. acidocaldarius*. The nature of biofilm formation by *S. acidocaldarius* was also strongly influenced by Aap pili. Biofilms of strains unable to make Aap pili were flat and denser than those of wildtype cells and they lacked the tower structures observed in the biofilms of wildtype cells [9,32]. 

Studies aimed at elucidating a function for the pili of *M. maripaludis* demonstrated a role in attachment to various abiotic surfaces [31] but this role was co-dependent on the presence of archaella (see Section 3.6.). Since attachment of cells to surfaces and other cells was via bundles of archaella, it may be that the prolonged attachment is mediated by archaella and the pili role may be more inthe initial stages, which is exactly the opposite to the roles suggested for Ups pili and archaella in *S. solfataricus* [105]. 

In *Hfx. volcanii*, nonarchaellated mutants adhere as effectively as wildtype cells to glass cover slips indicating that archaella in this strain are not strictly necessary for attachment, at least under laboratory conditions [26]. However, a *pibD* deletion mutant did not adhere, indicating that another appendage, likely pili, composed of subunits processed by PibD, was responsible for attachment [26]. In addition, while Ups pili are known to lead to cell aggregation and subsequent DNA transfer in *Sulfolobus* species, type IV-like pilus structures are not involved in the conjugative transfer of DNA observed in *Hfx. volcanii* since the rate of conjugation is not affected when *pibD* is deleted [26]. However, formal identification of pili structures in *Hfx. volcanii* has not been reported, although a variety of filaments can be observed on the surface of *Hfx. volcanii* [26].

No evidence has been presented that suggests type IV-like pili in any archaeon are involved in surface motility (twitching), a common function of their bacterial counterpart. Twitching motility involves the extension and retraction of type IV pili through the activity of two separate ATPases, one to add subunits to the base of the structure (extension) and one to remove subunits (retraction) [108]. In archaeal type IV-like pili systems only a single ATPase has been identified, suggesting that retraction may not be possible.

## 5. Other Unusual Archaeal Surface Structures

### 5.1. Iho670 Fibers

**Figure 7:**
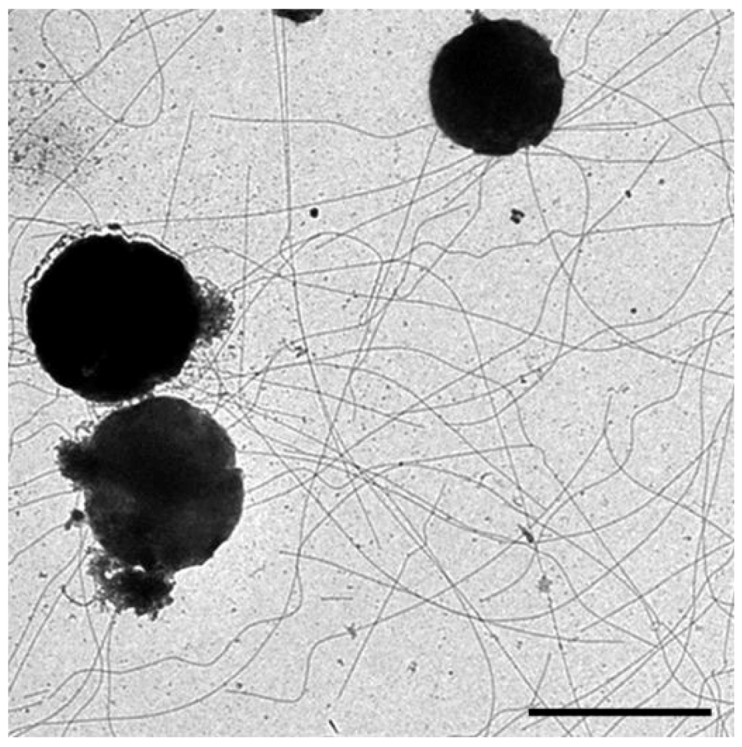
Electron micrograph of three *I. hospitalis* cells showing numerous Iho670 fibers on the carbon support film. Bar = 2 µm. Courtesy of Carolin Meyer and Reinhard Rachel, University of Regensburg, Germany.

A novel structure found in *Ignicoccus hospitalis *is the adhesive filament “Iho670 fiber”, so-called since they are comprised mainly of the protein encoded by the Igni_0670 gene [35]. These are extremely brittle and gentle handling of the cells must be employed in order to observe the appendages still attached to cells (Figure 7). The Iho670 protein was detected in appreciable amounts in a proteomic study regardless of whether *I. hospitalis *was grown in single culture or in co-culture with *Nanoarchaeum equitans, *an organism with which it can form an “intimate association” or biocoenosis [116,117]*. *Other than the hydrophobic amino terminus typical of all archaeal type IV pilin-like proteins, the Iho670 protein shows no primary sequence similarity to archaellins, the Mth60 fimbrin of *M. thermoautotrophicus*, the hamus protein of SM1 euryarchaeon or the three cannulae proteins of *Pyrodictium. *It does have a type IV pilin-like signal peptide (Table 1), confirmed by N-terminal sequencing of the mature protein [35].This signal peptide removal, detected by an *in vitro* processing assay, is likely carried out by the single prepilin peptidase (Igni_1405) detected in the completely sequenced genome. Iho670 fibers have a diameter of 14 nm and can be up to 20 µm in length; SDS-PAGE indicated the structure is composed of a single protein of 33 kDa [35]. The Iho670 protein has the potential to be glycosylated as *I. hospitalis* does have a putative oligosaccharyltransferase (Igni_0016; [118]) and the Iho670 protein does possess potential N-linked glycosylation sites. However, Iho670 proteins did not test positively for glycosylation when stained with the PAS reagent, although false negative results have been reported using this method. Structural analysis has revealed that single α-helices form the core of the Iho670 filament. The overall helical symmetry is similar to that of the archaellar filaments of *H. salinarum *and *S. shibatae,* however the quaternary structure of Iho670 fibers is, again, unique [10]. The fibers were also observed to transition between rigid and curved segments down the length of the filament, however the mechanism of switching and supercoiling has not yet been studied.

### 5.2. Cannulae

**Figure 8:**
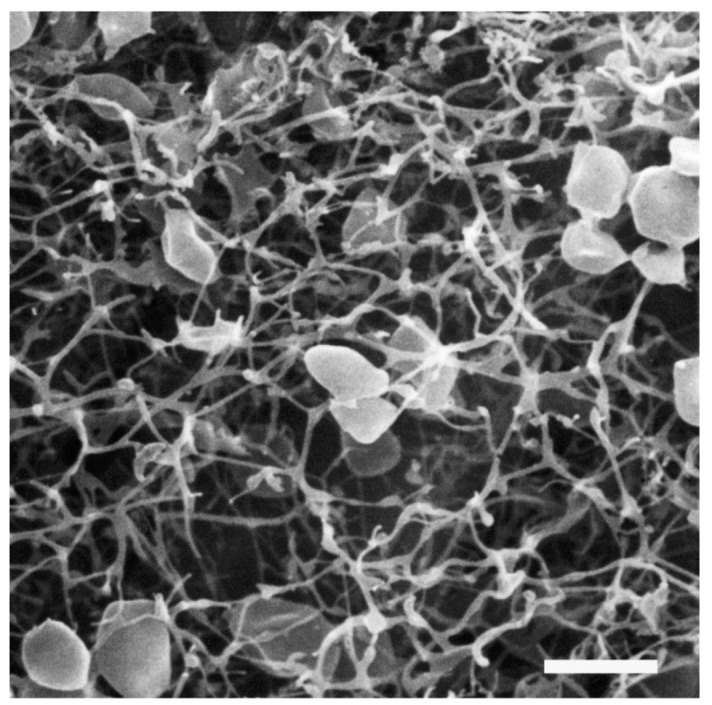
Scanning electron micrograph of a network of *Pyrodictium* cells and cannulae. Bar = 2 µm. Courtesy of Gertraud Rieger & Reinhard Rachel, University of Regensburg, Germany, and René Herrmann, ETH Zürich, Switzerland.

A distinctive feature of species of the hyperthermophilic genus *Pyrodictium *is that individual cells grow in a network of extracellular tubules called cannulae (Figure 8) [8]. Cannulae have an outer diameter of 25 nm and appear empty when thin-sectioned or cross-fractured. The cannula structure itself is composed of at least three related glycoproteins, termed CanA, CanB and CanC, with molecular masses in the 20–24 kDa range. The N-terminal 25 amino acids of each protein are identical [8] and Can proteins were reported to contain signal peptides [119]. The genes encoding the three cannulae protein subunits (*canA, canB *and *canC*) were identified [119] and later published in a patent application [120]. The overall cannula structure is remarkably heat resistant and insensitive to denaturing conditions; no loss of structure was observed even after incubation at 140 °C for 60 min or at 100 °C in 2% SDS for 10 min [8].

Three dimensional reconstructions of cannula-cell interactions provided the first evidence that cannulae enter the “periplasmic space” but not the cytoplasm of connected *Pyrodictium* cells [119]. Though it has been observed that cannulae elongate, remain attached as *Pyrodictium* cells and undergo binary fission [121], it remains unclear if any material, genetic or nutritive, is actually exchanged between cells through the cannula-cell connections. Cannulae may be necessary for the growth of *Pyrodictium *cells as spontaneous cannulae-free mutants have not been observed in laboratory cultures [119]. The functions of cannulae are unknown although a role in adhesion would not be unexpected [4]. Investigations into possible functions as well as the mechanism of assembly are hampered by the lack of a genetic system in *Pyrodictium* and an inability thus far to obtain strains unable to synthesize the tubules. Nevertheless, the massive amounts of material devoted to the structures point to an important role for these appendages.

### 5.3. Hami

**Figure 9:**
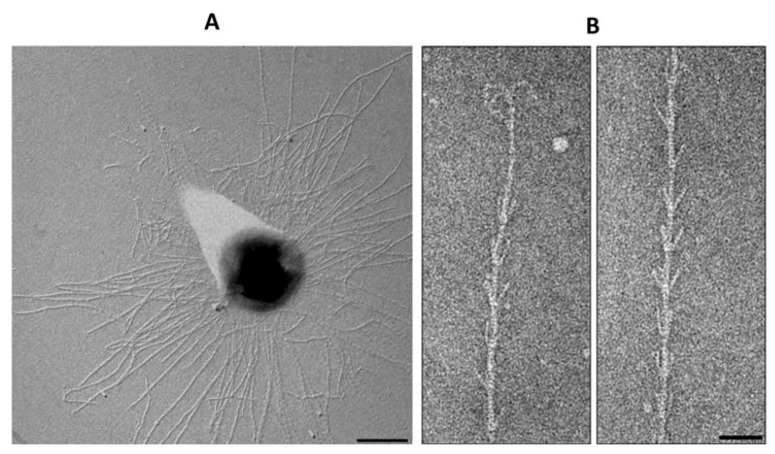
The hami of euryarchaeon SM1. (**A**) Electron micrograph of SM1 cells with numerous hami on the surface. Bar = 500nm. (**B**) The hook and prickle region of a hamus filament. Bar = 50 nm. Courtesy of Christine Moissl-Eichinger, Institute for Microbiology and Archaea Center, Regensburg.

Studies of Archaea in non-geothermal environments revealed the existence of the SM1 euryarchaeon whose filamentous “hami” represent another cell surface appendage unique to Archaea. The term “hamus” (Latin for hook, barb) is a direct reference to this surface appendage’s characteristic structure as each pilus-like fiber ends in a three-pronged tip [7]. The archaeal cells exhibiting hami grow in cold (~10 °C) sulphidic springs as members of archaeal/bacterial communities with mainly *Thiothrix* or IMB1 ε-proteobacterium as the bacterial member [121,122]. These archaeal/bacterial communities resemble a string-of-pearls that are macroscopically visible, as the whitish pearls can reach a diameter of up to 3 mm. The SM1 organisms are small cocci, with approximately 100 filamentous hami emanating peritrichously from each cell (Figure 9A). Hami are 7 to 8 nm in diameter and 1 to 3 µm long; the filament structure is helical with no evidence of a central channel. Three prickles (4 nm in diameter) radiate from the filament every 46 nm, giving the filament the appearance of barbwire. Distally, the filament ends with a tripartite hook (diameter 60 nm); with its thicker ends (diameter 5 nm), this hook region is likened to grappling hooks and anchors (Figure 9B) [7]. The hamus structure remains stable across a wide temperature and pH range (0–70 °C; pH 0.5–11.5), and is formed by a 120 kDa protein. While potential glycosylation of the protein was investigated, it did not stain with the PAS reagent or digest with PNGaseF (Peptide N-glycosidase F). However, neither test rules out conclusively the presence of attached glycan. Hami facilitate a strong adhesion of individual SM1 euryarcheon cells to chemically diverse surfaces as well as to their bacterial partners in sulphidic springs [7,123].

Though the SM1 euryarcheon has withstood attempts at laboratory cultivation, biofilms predominantly consisting of SM1 euryarchaea were harvested from a sulphidic spring near Regensburg, Germany. The opaque, white droplets of SM1 biofilm collected on polyethylene nets were decidedly different from previously characterized SM1/bacterial string-of-pearls communities; the harvested SM1 biofilms predominantly consisted of archaeal cells (>95%) whereas the string-of-pearls communities had archaea to bacteria in ratios closer to 1:1. Examined under confocal laser scanning microscopy, the SM1 cells in the biofilm were observed to be approximately 4 µm apart, surrounded by an extracellular polymeric substance (EPS) composed of proteins and polysaccharides. The regular separation of single cells is thought to be the result of contact between the hami of neighboring cells, whose average length of 2 µm corresponds to the cell-cell distance of 4 µm. Hami also contribute to the EPS as its main protein component; as hami filaments entangle, they create a web between cells which contributes to the overall biofilm structure. The function of hami as mediators of cell-surface attachment and biofilm initialization has been proposed [124] as a variation of the role often played by pili and/or flagella in the formation of many bacterial biofilms.

### 5.4. Bindosome

The bindosome is a surface structure of *S. solfataricus *comprised of sugar binding proteins which act with ABC transporters to facilitate sugar uptake [125,126]. The sugar binding proteins GlcS and AraS have type IV pilin-like signal peptides which are processed by PibD, a type IV prepilin-like peptidase of *S. solfataricus* [25] . The expression of GlcS and AraS in the cell surface is directed by the bindosome assembly system (Bas), yet another type IV pilus like assembly system is composed of three pilin proteins BasABC, BasE, a PilT-like ATPase, and BasF, a PilC-like integral membrane protein [43]. Deletion of either of these gene groups resulted in severe defects in the ability of the cells to grow on sugars transported by these sugar binding proteins. 

Since the Bas system appears similar to the bacterial type IV pilus assembly system, it was suspected to result in a pilus-like structure. While such a specific bindosome appendage has not been observed on cell surfaces, it is believed that a sugar binding structure embedded into the cell wall is integral to the *S. solfataricus* envelope [5]. It was recently reported that the ATPase activity of BasE is required for glucose uptake, and that the sugar binding proteins occur in complexes of high molecular mass. SlaA, the S layer glycoprotein, was also identified in these complexes, suggesting that the sugar binding proteins in *S. solfataricus* associate with the S-layer. On a cellular level, the deletion of *basEF* resulted in abnormal morphology and S-layer architecture; this further suggests that the sugar binding proteins are a functional part of the S-layer in *S. solfataricus* [127].

While studies are so far limited to *S. solfataricus*, bindosomes may actually be widely distributed in Archaea since many substrate binding proteins with type IV pilin-like signal peptides can be identified in both crenarchaeotes and euryarchaeotes [24]. 

## 6. Concluding Remarks

Archaea possess a wide variety of surface structures that contribute to their survival in what are often extremely harsh environmental niches. This requires that these structures be extremely stable to such stresses as high salt, extremely high temperatures and low pH. The mechanisms by which this occurs are currently unknown but may be partially due to the often encountered N-glycosylation of the major subunits of the structures. The Archaea have adapted the type IV pilus model for use in assembly of a diverse repertoire of appendages performing a variety of functions including swimming and adhesion. Some of the most unusual and abundant of the surface structures, like hami and cannulae, are currently only found on Archaea that lack a developed genetic system. Thus how these complex structures are formed, let alone the full listing of their functions, remains a mystery. Continued study of archaeal surface structures will undoubtedly provide a much needed insight into how archaeal cells thrive in their unusual habitats and how they interact with their neighbors and abiotic surfaces. Elucidation of their assembly mechanisms will lead to new discoveries about regulation in archaeal systems. This has recently begun in studies showing the involvement of regulators in the biosynthesis of archaella and pili in *Sulfolobus*. Studies on the roles of posttranslational modifications of surface structure components may shed light on protein stability enhancement via glycosylation and lead to biotechnological advances. As more Archaea become genetically tractable, new discoveries will be made in currently less well-studied, but no less interesting, members of the domain. 

We recommend that readers also consult a recent review by the Sonja Albers group [128] which was published during the final stages of preparation of this review. 
